# The Fate of the Tumor in the Hands of Microenvironment: Role of TAMs and mTOR Pathway

**DOI:** 10.1155/2016/8910520

**Published:** 2016-12-15

**Authors:** Danilo Figueiredo Soave, Marina Pacheco Miguel, Fernanda Dias Tomé, Liliana Borges de Menezes, Patrícia Resende Alo Nagib, Mara Rúbia Nunes Celes

**Affiliations:** ^1^Department of Histology, Embryology and Cell Biology, Institute of Biological Science, Federal University of Goias, Goiania, GO, Brazil; ^2^Department of Microbiology, Immunology, Parasitology and Pathology, Institute of Tropical Pathology and Public Health, Federal University of Goias, Goiania, GO, Brazil; ^3^Department of Pathology, Faculty of Medicine of Ribeirao Preto, University of Sao Paulo, Ribeirao Preto, SP, Brazil

## Abstract

Since 2000, written with elegance and accuracy, Hanahan and Weinberg have proposed six major hallmarks of cancer and, together, they provide great advances to the understanding of tumoral biology. Our knowledge about tumor behavior has improved and the investigators have now recognized that inflammatory microenvironment may be a new feature for the tumor entities. Macrophages are considered as an important component of tumoral microenvironment. Biologically, two forms of activated macrophages can be observed: classically activated macrophages (M1) and alternative activated macrophages (M2). Despite the canonical pathways that control this puzzle of macrophages polarization, recently, mTOR signaling pathway has been implicated as an important piece in determining the metabolic and functional differentiation of M1 and M2 profiles. Currently, it is believed that macrophages related to tumoral microenvironment present an “M2-like” feature promoting an immunosuppressive microenvironment enhancing tumoral angiogenesis, growth, and metastasis. In the present review we discuss the role of macrophages in the tumor microenvironment and the role of mTOR pathway in M1 and M2 differentiation. We also discuss the recent findings in M1 and M2 polarization as a possible target in the cancer therapy.

## 1. Introduction

Today there is a great debate about the role of inflammation in control and regulation of tumor microenvironment directly influencing the development of neoplasms. Inflammation plays a fundamental role in tumor dynamic and is accepted as one of the hallmarks of cancer [1, 2]. Currently there are two marked ways by which inflammation is associated with cancer development, an intrinsic pathway characterized by the gene expression and an extrinsic pathway, characterized by the formation of an inflammatory microenvironment [3]. Within this microenvironment, macrophages are the major cell populations involved in the inflammatory process associated with the development of neoplasms [4]. Considered highly plastic cells, they are able to respond to stimuli and produce numerous pro- and anti-inflammatory factors and when related to cancer, they are termed tumor-associated macrophages (TAMs) [5].

It is believed that TAMs play a key role in promoting and coordination phenomena of tumor growth due to their ability to modulate the angiogenesis and lymphangiogenesis that are processes involved in the progression of neoplasms [6]. TAMs are activated by different stimuli, such as growth factors, nutrients, and cytokines, produced in the tumor microenvironment that are responsible for inducing differentiation in functionally distinct populations. In populations of classically activated macrophages (M1), there is a high production of proinflammatory cytokines displaying an immune-stimulatory function. On the other hand, the alternative activation of macrophages (M2) is based on the release of anti-inflammatory cytokines supporting an immunosuppressive environment [7–9].

Thus, the microenvironment plays dual antagonist roles in tumors. This environment, formed by cells, extracellular matrix, growth factors, nutrients, and cytokines, may be responsible for the macrophages differentiation and behavior of tumor cells. However, like a feedback loop, these cells alter the environment in reply to stimuli. In addition, network interactions can define the development of tumors. In this way, the role of TAMs polarization in M1 and M2 profiles might have clinical and pathological importance and appears to be associated with angiogenesis, proliferation, aggressiveness of tumor, and apoptosis. Moreover, the debate appears to be controversial about the relation between inhibitory or promoter ability of M1 or M2 on tumor [10].

Recently, one of the ways suggested for activation of macrophages subpopulations involves the signaling Akt/mTOR pathway. A change in regulation of this metabolic pathway plays a crucial role in activation controlling and acquisition of macrophages effector activity, depending on the context in which they are, as well as the tumor microenvironment [11]. With this view of the tumor stroma and its influence on the progression of neoplasms, the study about the role of macrophage polarization in the tumor pathogenesis may emerge as a new therapeutic approach. In this review, we discuss the role of tumor microenvironment and macrophages in cancer; M1 and M2 differentiation and the role of mTOR pathway; and M1 and M2 macrophages as possible tumor markers.

## 2. The Macrophages and Tumor Microenvironment

The monocyte-derived macrophages are cells of the myeloid lineage that have functional plasticity [12, 13]. They are important cells of the innate immune system and can act in different tissues as phagocytes, antigen presenting cells, and tissue remodeling [14, 15]. The functional plasticity associated with phenotypic and metabolic differences led to the characterization of two macrophage subtypes, M1 and M2. These macrophages subtypes were primarily defined in murine models, but actually the M1 and M2 terms are widely used for humans and others mammalians. However, clearly, there are not only two functional subtypes of macrophages and the polarization process has a large spectrum of subpopulations with phenotypic differences showing the plasticity of macrophages [15, 16].

M1 macrophages are always related to the inflammatory response by presenting a large phagocytic and microbicidal capacity, increased expression of MHC II molecules, CD80, and CD86 and also secreting cytokines such as TNF-alpha. This M1 profile is induced by the presence of PAMPs that bind mainly to TLR2 and TLR4 and Th1 cytokines such as IFN-gamma that activate the transcription factors STAT1 and IRF5 [12, 14, 15].

M2 macrophages, in turn, are typically activated in the presence of Th2 cytokines such as IL-4 and IL-13 that improve the STAT6 and IRF4 interaction. The M2 profile is characterized by low phagocytic and microbicidal activity, and it is linked to the presence of helminthes or to the tissue remodeling processes. These M2 macrophages, unlike the M1, do not activate iNOS and therefore do not produce nitric oxide (NO); however, they have great arginase activity that is important in wound healing and tissue remodeling process [14, 16]. The differences between these profiles are also observed in their metabolism, M1 have preferably glycolytic pathways while M2 has increased beta-oxidation [11, 12].

TAMs are a major stromal component and exhibit, mostly, M2 similar profile. However, according to Sica and Mantovani (2012) macrophages change during tumor development. TAMs show M1-like phenotype characterized by high IL-12 releases that cause tumor cell disruption, when in tumoral early-stage. Despite this, in late-stage of tumor progression, TAMs exhibit an M2-like phenotype accompanied to reduced antitumoral activity [17].

The antitumoral M1-dependent response is related to inflammatory response and the activation of specific lymphocytes in an attempt to eliminate tumor cells. On the other hand, M2 participation in tumor environment provides angiogenesis and survival, supporting metastasis and tumor growth [11, 18].

Thus, TAMs play a crucial role in the modulation of tumor microenvironment producing a large amount of growth factors and inflammatory mediators that stimulate proliferation, angiogenesis, deposition, or degradation of extracellular matrix [19, 20]

## 3. Angiogenesis and Lymphangiogenesis

The tumor cells present an unusual metabolic activity and consume a large amount of nutrients and oxygen; in addition, these cells need to remove the waste metabolic products such as carbon dioxide. Thus, the new vascularization associated with tumor, generated by the angiogenesis process, allows these conditions [21]. Angiogenesis is essential for the survival, development, and progression of tumor [22]. Thus, without a pathological neovascularization the tumors grow slower decreasing their capacity to promote metastasis. At the same time, ineffective blood supply does not allow the action of anticancer drugs in effective amounts in tumor regions [23].

In some cancers the angiogenesis stimuli may occur in two ways. The first occurs directly by oncogenes such as Ras and Myc that can increase the expression of angiogenic factors in cancer cells, while, in second, the angiogenic signals are indirectly produced by inflammatory cells, specially TAMs [21] that play a crucial role in tumor promotion and progression, contributing to neovascularization and tumor cell proliferation and metastasis [24–27]. The epidermal growth factor (EGF); family members of fibroblast growth factor (FGF); VEGF; matrix metalloproteinases (MMPs); heparanase; plasmin, activator of the urokinase-type plasminogen (uPA); the uPA receptor; and cytokines and chemokines as TGF-*β*, which enlarge the inflammatory state, are among the key related factors secreted by TAMs in the tumor microenvironment [21, 25, 28].

Proangiogenic factors produced by TAMs are critical to regulate tumor development and have ability to maintain a favorable environment to encourage and maintain the remodeling and a complex vascular network within the lesion [29]. VEGF [9] and matrix metalloproteinase-9 zymogen (proMMP-9) [30] are considered key factors involved in this increased organization of tumor vasculature. Furthermore, Zajac et al., 2013, demonstrated that the angiogenic capacity of M2 macrophages critically depends on production of the readily activatable proenzyme proMMP-9 and only M2 macrophages approach the high angiogenic capability of neutrophils [30].

In addition to angiogenesis researchers have also studied a new approach in vascular biology “the lymphangiogenesis.” Lymphangiogenesis is described as the development of a new lymphatic vessels [31], which in normal condition occurs in embryonic development being absent in adults. However, this process can be induced by pathological condition such as chronic inflammation, wound healing, and several neoplastic lesions [31, 32]. As angiogenesis, the process of lymphangiogenesis can be stimulated by cytokines and growth factors; the Vascular Endothelial Growth Factor family (VEGFs) plays a pivotal role in this mechanism. Related to lymphangiogenesis, VEGF-C and VEGF-D present an exclusive involvement and recent data has also described VEGF-A, a well known angiogenic growth factor, as an important lymphangiogenic growth factor [33, 34]. The expression of lymphangiogenic growth factor and related protein was revised by Ran and Montgomery (2012) and, together with VEGF, the authors listed PDGF, adrenomedullin, HGF, COX-2, FGF, TNF-alpha, MMPs, heparanase, and angiopoietin-2 as factors that can also provide lymphangiogenesis [35].

Ruffell et al. (2012) describes the VEGF production not only by tumor cells but also by TAMs [9] and this starts the lymphangiogenic process through VEGF-C and VEGF-D via VEGFR3 [36] Results presented on cancers of the cervix postulate the hypothesis of peritumoral lymphangiogenesis process mediated by TAMs. In first step, the macrophage population is chemoattracted and triggered to TAMs phenotype toward the neoplastic microenvironment. Thus, activated TAMs serve as a major producer of VEGF-C and VEGF-D that cause proliferation of lymphatic microvessels and this new dense vessel network could favor the lymphatic metastases formation [36].

Once recruited into neoplastic microenvironment TAMs may also disrupt lymphangiogenesis and lymphatic tumoral spread in pancreatic cancer; Kurahara and colleagues (2011) reported that this tumor presented a high density of TAMs correlated with lymph node metastasis. Additionally, these authors also analyzed the correlation between CD163+ densities with clinic-pathological data, and, as a result, the “M2-like” phenotypes are correlated to more aggressive neoplasm entities [37]. The evidences that macrophages can cooperate with lymphangiogenesis also came from studies on bladder cancer [38], breast [39], lungs [40], and melanoma [41]. The research data presented above suggest that macrophages induce the formation of both angiogenic and lymphangiogenic microenvironment.

In contrast, these cells can also express antiangiogenic molecules and damage the integrity of the blood vessels [28]. This paradoxical role of M1 and M2 in cancer is explained by their functional plasticity, which results in the expression of pro- or antitumor functions [24, 42, 43].

The antiangiogenic effect has been demonstrated in colonic carcinoma after using tasquinimod. The treatment induced an antitumor effect by subsequent reduction of M2 CD206+ macrophages and a simultaneous increase in M1 macrophages expressing class II MHC and CD86+. This change was preceded by an increase in IL-12 production within the tumor and a reduction in its neovascularization [44]. Takeuchi et al. (2016) to correlate the prevalence of M2 macrophages, angiogenesis, and clinic-pathological features in bladder cancer showed higher density of M2 macrophages in invasive bladder cancer in relation to non-muscle invasive cancers. Moreover, the higher the distribution of microvessels, the greater the degree of severity of cancer, and the predominance of M2 macrophage correlated to the amount of microvessels in bladder cancer and can have prognostic value for this type of cancer [45].

Although high concentration of M2 promotes tumor angiogenesis, the mechanism that promotes the differentiation of monocytes in TAMs and their phenotypes is still unclear. Thus, it was proposed that the mTOR pathway could be a critical element in the regulation of TAMs differentiation [45].

## 4. M1 and M2 Differentiation and the Role of mTOR Pathway

The molecular control of polarization and activation of M1 and M2 has been the subject of many investigations and has well established the participation of STAT1 and STAT6, respectively [13, 16]. In addition to these canonical pathways, molecules of other routes are also being related to differentiation of macrophages [11, 46].

Mammalian target of rapamycin (mTOR), a serine-threonine kinase that is highly conserved, is a component of the pathway cell survival phosphatidylinositol 3-kinase (PI3K) that monitors the availability of nutrients, mitogenic signals, cellular energy, and oxygen levels and is therefore insignificant in the regulation of cell growth and proliferation [11, 47, 48]. mTOR is regulated by nutrients and growth factor and regulates cell growth by controlling many biological process such as autophagia, mRNA translation, and energetic metabolism [Guertin and Sabatini, 2007] [49].

The mTOR acts both upstream and downstream of the Akt, operating as a connection with PI3K pathway and forming two different multiprotein complexes, mTORC1 (mTOR complex 1) and mTORC2 (mTOR complex 2), which regulate protein synthesis required for cell growth and proliferation [48, 50]. The stimulation of TLRs by PAMPs leads to the recruitment of PI3K that activates mTORC1 and mTORC2 in macrophages [47].

The mTORC1 may be inhibited physiologically by the action of TSC1 (tuberous sclerosis complex 1) and TSC2 (tuberous sclerosis complex 2) and by rapamycin due to the presence of Raptor (regulatory associated protein of mTOR) that is sensitive to the action of rapamycin [48, 50–53]. Mutations in TSC1 or TSC2 genes lead to tuberous sclerosis syndrome with the appearance of various benign tumors in skin, kidney, brain, heart, and lung [48, 50, 54]. mTORC1 is described as a regulator of the inflammatory activity of macrophages and other innate cells because its activation is related to reduction of the NF*κ*B activity and increase of IL-10, TGF-*β*, and PDL1 expression [47].

mTORC2, on the other hand, is insensitive to rapamycin and Rictor (rapamycin insensitive companion of mTOR) and Sin1 are two specific proteins for this complex. Moreover, the mTORC2 can be activated by specific growth factors in macrophages and phosphorylates AKt. The specific mTORC2 function in macrophages remains unclear, but recent studies showed that since the lack mTORC2, the inflammatory response is augmented in dendritic cells [55] and macrophages [56]. In this study with murine macrophages lacking Rictor, the authors demonstrated the overresponse to TLRs ligands and the increase of the levels of M1 related-genes and lower expression of M2 markers. These data suggest that mTORC2 plays key roles in the macrophage polarization and in the regulation of the inflammatory response [56].

In 2013, a group of researchers using rapamycin in human monocytes showed that monocytes had a cytokine profile after stimulation similar to that expected for M1 [53]. In the same year, Byles and colleagues showed that the TSC1-mTOR pathway regulated the polarization of human macrophages. They showed that, in the absence of TSC1, the macrophages did not differ to M2 even in the presence of IL-4 and suggested that activation of mTORC1 would lead to downregulation of Akt interfering with the expression of characteristic M2 genes [54]. In addition, recently, Huang and collaborators (2015) observed that glycolytic metabolism, specific of M2 profile, requires both mTORC2 and Stat6 pathway activation in IL-4 and CSF1-stimulated macrophages [52]. Lin et al. (2002) highlighted the CSF-1 as one of the major regulators to mononuclear cells and its expression in breast carcinomas is correlated with poor prognosis [57].

In 2014, Zhu and colleagues also related the macrophages polarization to TSC1 and mTOR. In this case, the authors used TSC1 knockout mice and observed resistance to M2 profile polarization and M1 macrophages increased. However, they concluded that M2 inhibition occurred in mTOR-dependent manner and the M1 increase was mTOR-independent and ERK-dependent [58]. Additionally, mTOR pathway is also responsible for the control of cellular metabolism, which appears differently between the poles [11].

In the tumor context, TAMs also have their activities regulated by TSC-mTOR complex. Chen et al. (2012) demonstrated that, in human peripheral monocytes stimulated by lipopolysaccharide, the mTOR was inhibited by rapamycin or activated by RNA interference-mediated knockdown of the mTOR repressor tuberous sclerosis complex 2 (TSC2) [59]. Thus, rapamycin took to differentiation of monocytes into macrophages M1 releasing more IL-12 and less IL-10 while TSC2 knockdown led to the differentiation of monocytes into macrophages M2 releasing less IL-12 and more IL-10. Moreover, angiogenic properties were stimulated from endothelial cells from human umbilical vein cocultured with TSC2 deficient monocytes or reduced in the group treated with rapamycin. The tumor angiogenesis and growth in murine xenografts were held after infusion of TSC2 deficient monocytes or reduced with monocytes overexpressing TSC2. Finally, in vivo depletion of macrophages was sufficient to block the antiangiogenic effects of rapamycin in tumors. These results define the TSC2-mTOR pathway as a determinant in the differentiation of monocytes in TAM M2 phenotype that promote angiogenesis. [59].

In a recent paper, Yang et al. (2016) demonstrated that macrophages, associated with the microenvironment of the renal cell carcinoma, favor tumor metastasis by activation of Akt/mTOR pathway [60].

In addition, as for the mTOR signaling to be related to macrophages differentiation and consequently in angiogenesis, studies show its role in proliferation, migration and cell survival [59, 61, 62]. These data together reinforce that the mTOR pathway can be used as a therapeutic target in an attempt to modulate macrophage response in the tumor environment or the raised immune response so that there is an antitumor effect.

## 5. mTOR Activation in Cancer Cell

Not only macrophages but also cancer cells present mTOR pathway activation that modulates different molecules that contribute to the tumoral microenvironment. In turn, this environment can induce the TAM polarization, demonstrating the close and complex relationship between cancer cell, macrophages, and tumor progression.

Gene mutations such as oncogenes and tumor suppressor genes confer constitutive activation of the mTOR pathway in conditions of nutrient depletion increasing its expression in various types of cancer, such as AKT in breast and ovarian cancer, PI3K in ovarian, gastrointestinal, breast, and prostate cancer, TSC1/TSC2 in the hamartomas formation and lymphangioleiomyomatosis development [63]. However, the inhibition by rapamycin or nutrient depletion induces the activation of autophagy by PI3 kinase, AKT, and mTOR pathways, which allows the cancerous cells survival under conditions of a microenvironment with low amount of nutrients [59, 63]. In autophagy, the release of amino acids arising from autophagic degradation generates reactivation of mTORC1 and the restoration of lysosomal cell population and promotes cell growth [61].

mTOR pathway plays a critical role in the tumoral neovascularization providing an environment where cells grow. Initially, tumor microenvironment modifications can induce mTOR to increase the translation of hypoxia-inducible factor 1 (HIF-1)/hypoxia-inducible factor 2 (HIF-2), which drive the expression of genes for hypoxic stress response, including angiogenic growth factors such as VEGF, PDGF-*β*, and TGF-*α* [64]. Interestingly, VEGF has also presented the direct capability to activate the mammalian target of rapamycin complex 1 (mTORC1) pathway through the cross talk with phospholipase C gamma (PLC*γ*). As mTORC1 is a metabolic sensor, metabolic signals may be integrated with signals from VEGF in the regulation of angiogenesis leading to a positive feedback loop to enhance angiogenic responses [65].

Furthermore, the lymphangiogenesis can be also controlled by mTOR pathway modulation. To illustrate the relation between mTOR and lymphangiogenesis Ekshyyan et al. (2013) demonstrated an association between mTOR inhibitor and tumoral growth. The authors observed that rapamycin (mTOR inhibitor) significantly inhibited tumor progression and lymphangiogenesis when compared to control, and in conclusion Ekshyyan et al. (2013) describes mTOR inhibitor impairing the autocrine and paracrine VEGF-C/VEGFR-3 axis. The Ekshyyan et al. (2013) conclusion could be linked to TMAs population; mTOR inhibitor can affect macrophages changing their expression profile [66]. Corroborating this idea Lee et al. 2010 describes a downregulation of proinflammatory mediator related to rapamycin treatment [67].

## 6. Macrophages, Survival, and Prognostic Factor

In last decades significant improvement of overall survival has been observed in patients with neoplastic disease. Some factors (locoregional recurrences, distant metastases, and a second primary tumor) may influence the prognosis reducing overall survival rates. In recent years, a better understanding of tumor-associated macrophages associated with these tumors microenvironment might help to find adjuvant therapies to improve patients' overall survival.

Several studies have now established that tumor-associated macrophages should influence tumoral prognoses. We need to remember that TAMs may present an M2-like (immunosuppressive) phenotype that leads to enhancing the tumor development. However, some cite that an M1-like (proinflammatory phenotype) population predominate [3]. Therefore, the TAMs phenotype may play a dual role in tumoral progression and disease overall survival. An animal model of lung cancer demonstrated that the immunomodulation of M2 TAMs population was able to reduce the TAMs population and tumor size and enhances overall survival rates [68]. An analysis performed by Osinsky et al. (2011) aimed at analyzing the impact of tumoral microenvironment on survival outcomes showed that patients diagnosed with human gastric cancer that have high level of TAMs presented a significantly lower overall survival when compared to patients presenting low level of TAMs [69].

In a retrospective study comparing TAMs population in pancreatic ductal adenocarcinoma Chen et al. (2015) described that tumor presenting a high level of TAMs infiltration has a significantly worse relation to prognostic factors and lower survival rates [70]. As a first analysis the authors observed distribution of CD68 and CD163 (M2-like macrophages), and focused on M2 phenotype the authors observed a positive relation to histological grade and N stage. However, CD163 macrophages were related to smaller tumors. Chen et al. (2015) have also demonstrated the relation between TAMs and survival; neoplasm with high M2-like macrophage infiltration presented a worse survival rate when compared to lesion presenting a low M2-like infiltration [70].

Corroborating the idea of worse prognoses of M2-infiltrating TAMs a study evaluating the correlation between CD163+ macrophages and survival rates in melanomas showed that lesions presenting a high population of CD163+ macrophages have significantly poor overall and melanoma-specific survival [71]. In the same way, breast cancer has also demonstrated a worse prognosis related to TAMs infiltration, and Tsutsui et al. (2005) described that patients with high TAMs population have a reduced disease-free survival [72]. This relation has also been observed in kidney cancer. However, this behavior (relation between TAMs and worse prognoses) is not rule for all neoplastic entities. To exemplify the correlation between TAMs infiltration and better prognosis, Edin et al. (2012) studying colorectal cancer demonstrated a significant association between improved prognosis and CD163+ TAMs [73]. To explain this relation the authors demonstrate the parallel existence of a NOS2+ TAMs population (M1-like macrophage), and Edin et al. (2012) believe that patient's outcome was determined by M1 and M2 phenotypes balance [73].

## 7. Conclusion

The tumor-associated macrophages have emerged as a main component effector and regulator of the innate immune response to neoplasms. Biologically, the cancer microenvironment has provided important signals to macrophages plasticity. In this context, the mTOR signaling pathway, a key factor in M1 and M2 polarization, can be, in the near future, considered as possible target in anticancer therapy.
